# Preliminary Pharmacokinetic Analysis of Tramadol and Its Metabolite O-Desmethyltramadol in Boa (*Boa constrictor constrictor*)

**DOI:** 10.3390/ani15162404

**Published:** 2025-08-15

**Authors:** Marina Lopes Castro, Natalya Maldonado Moreno, Raphael Rocha Wenceslau, Fabiola Paes Leme, José Eduardo Gonçalves, Lara Duque Estrada Meyer Fagundes, Natália Fagundes, Marcelo Pires Nogueira de Carvalho, Suzane Lilian Beier

**Affiliations:** 1 Department of Clinical and Veterinary Surgery, Veterinary School, Universidade Federal de Minas Gerais (UFMG), Belo Horizonte 31270-901, MG, Brazil; nina.lpes@gmail.com (M.L.C.); natalyamaldonadomoreno@gmail.com (N.M.M.); rwenceslau@hotmail.com (R.R.W.); fabiolaufmg@gmail.com (F.P.L.); marcelopnc@yahoo.com.br (M.P.N.d.C.); 2 Pharmacy Faculty, Universidade Federal de Minas Gerais (UFMG), Belo Horizonte 31270-901, MG, Brazil; dugoncalvesj@gmail.com; 3 Veterinarian Breeding Facility “Jiboias Brazil”, Betim 32670-402, MG, Brazil; larameyer.vet@gmail.com; 4 Institute of Molecular Biology, Graz University, 8010 Graz, Austria; nataliafagunds@gmail.com

**Keywords:** pain, reptiles, analgesia, opioids

## Abstract

*Boa constrictor* snakes represent a suitable model for studying the absorption, metabolism, and elimination of tramadol due to their distinct physiological characteristics. The objective of this work was to provide preliminary data on the pharmacokinetics of tramadol and its active metabolite, O-desmethyltramadol (M1), in the plasma of *Boa constrictor* using liquid chromatography with fluorescence detection. Ten snakes received tramadol (5 mg kg^−1^) both into the epaxial musculature (TRIM) and into the paravertebral vein (TRIV) with a 45-day interval between the two administration methods. Blood samples were taken at specified time points to analyze the pharmacokinetics. Data were evaluated with an independent pharmacokinetic model (R software version 4.3.0). A paired Student’s *t*-test was used for all parametric variables, except clearance, which was analyzed with the Wilcoxon test. A significance level of 5% was applied. The mean (range) maximum concentration of tramadol, volume of distribution, clearance, and elimination half-life for the TRIM group were 2.58 µg mL^−1^, 10.58 ± 2.91 L kg^−1^, 0.36 L kg^−1^ h^−1^, and 19.96 ± 8.34 h, respectively. For the TRIV group, these values were 3.39 µg mL^−1^, 5.60 ± 1.69 L kg^−1^, 0.22 L kg h^−1^, and 17.32 ± 7.55 h^−1^, respectively. M1 achieved maximum concentration and elimination half-lives of 0.58 µg mL^−1^ and 49.89 ± 10.8 h, respectively, for TRIM and 0.59 µg mL^−1^ and 35.66 ± 10.85 h for TRIV. The bioavailability of intramuscular tramadol was 61%, and M1 remained at similar concentrations for 20 min after tramadol administration in both treatments. Tramadol is rapidly biotransformed into M1 in *Boa constrictors*, maintaining high concentrations over an extended period. The pharmacokinetic characteristics, particularly the sustained plasma concentrations of M1, suggest potential for effective analgesia in the *Boa constrictor*. Furthermore, the intramuscular route provides the additional advantage of ease and practicality of administration.

## Simple Summary

Tramadol is commonly used for pain relief in both humans and animals; however, its pharmacokinetics in reptiles, especially snakes, are not well understood. Pharmacokinetics refers to how a drug is absorbed, distributed, metabolized, and eliminated by the body over time. Ten healthy male *Boa constrictor* were given tramadol through two different methods: intramuscular injection and direct intravenous administration. Pharmacokinetics were analyzed using a liquid chromatography model. Chromatography is a physical method used to separate the components of a sample in order to identify and quantify them. The results showed that when tramadol was injected into the muscle, it was absorbed moderately well, with a significant amount of the drug and its active form remaining in the snakes’ systems for a longer time. The observed sustained high concentrations of the active metabolite M1 suggest its potential as an analgesic in snakes. These findings suggest that tramadol could be an effective pain relief option for snakes. Understanding the pharmacokinetics of tramadol in these animals can help improve their health and welfare, ensuring they receive adequate pain management when needed.

## 1. Introduction

The increasing popularity of snakes, which are considered unconventional pets, has led to a growing demand for veterinary care. However, information on analgesics and pain treatments for these reptiles remains scarce [1]. Tramadol is considered a vital agent for scientific research due to its unique pharmacological properties and broad applicability in veterinary medicine [2,3]. Tramadol is an atypical opioid whose analgesic effect is primarily mediated by its metabolite O-desmethyltramadol (M1), which acts on µ opioid receptors in various animal species, and to a lesser extent by the inhibition of serotonin and norepinephrine reuptake. The µ receptors are located in the central nervous system and peripheral tissues of mammals and reptiles and are involved in supraspinal analgesia [2,3].

Tramadol has been used in veterinary medicine for its potential to alleviate mild-to-moderate pain, offering the advantage of causing minimal adverse effects on the respiratory, cardiovascular, and gastrointestinal systems in both humans and animals. Owing to these characteristics, this medication has become a recommended option for multimodal pain management and demonstrates versatility across various administration routes, including oral (PO), subcutaneous (SC), intramuscular (IM), and intravenous (IV) delivery [4,5]. Given its efficacy and favorable safety profile, tramadol plays an important role in managing pain in reptiles, a field where therapeutic options remain notably limited [4,5,6]. Pharmacokinetic studies are fundamental for elucidating the disposition of drugs across different species, making it essential to account for interspecies variability and the methodologies employed in various investigations [1]. In snakes, no information is available regarding the pharmacokinetics and its active metabolite M1. A dose of 5 mg kg^−1^ through IV and IM routes of administration has not been reported in snakes for this drug, highlighting the importance of investigating it in these animals. This study represents a pioneering application of liquid chromatography with fluorescence detection for the analysis of snake plasma, thereby contributing to a deeper understanding of the pharmacokinetics of tramadol in this species [1,2]. The primary objective of this research was to delineate the pharmacokinetics of tramadol and its active metabolite, M1, in the plasma of *Boa constrictors* utilizing this analytical technique. Given the established versatility and safety profile of tramadol, this investigation aims to further substantiate its potential as a reliable and effective analgesic for reptilian species.

## 2. Materials and Methods

This study is an experimental crossover.

### 2.1. Chemicals

Tramadol hydrochloride, its M1 metabolite, and ondansetron hydrochloride were purchased from Sigma-Aldrich (St. Louis, MO, USA). High-pressure liquid chromatography (HPLC)-grade methanol, acetonitrile, triethanolamine, and phosphoric acid were also obtained from Sigma-Aldrich, while anhydrous disodium phosphate was supplied by a local manufacturer (São Paulo, Brazil).

### 2.2. Study Sample

Ten healthy male breeding *Boa constrictor* with good body condition and an average weight of 4.33 ± 1.5 kg (mean ± standard deviation) were selected for this study. The animals were sourced from a licensed breeding facility, which provided authorization for their use in accordance with ethical guidelines and applicable regulations.

This study was approved by the Ethics Committee for Animal Use under protocol 171/2021 and registered in the National System for Management of Genetic Heritage and Associated Traditional Knowledge (SisGen number A5BCF84), authorized by the Ministry of Environment through the Biodiversity Authorization and Information System (SISBIO number 78843-1), and Jiboias, Brazil (licensed by IBAMA/IEF) as a breeding facility.

### 2.3. Experimental Design

The sample size calculation determined that ten snakes per group would be sufficient to detect differences with a significance level of 5%. The expected standard deviation was assumed from data reported by Itami et al. [7], with a 20% margin of error. To ensure homogeneity, factors such as species, body weight, and reproductive maturity were considered. Inclusion criteria required animals to be healthy, as confirmed by physical examination and complete blood count. The sample size was determined based on the mean of the most unstable studied variable (area under the concentration curve from zero to infinity) using the confidence interval formula with a 5% type I error [8].

The animals were kept in individual plastic boxes measuring 63.1 × 44.1 × 33.1 cm with a capacity of 68 L, equipped with small holes for air circulation. A paper towel was used as the substrate and was replaced as required. Water was provided ad libitum in plastic containers (water dispensers designed for the dogs). The plastic boxes were labeled with the names of the animals. The diet consists of rodents (rats and mice) and birds, with feeding occurring once every two weeks.

Before the experimentation, the snakes were weighed, transferred, and kept in a well-ventilated room designated for experimentation, with the monitored temperature maintained within the optimal range for the species (between 28 and 30 °C).

This study employed a fixed-sequence crossover design involving ten animals, each of which received both tramadol treatments—first via intramuscular (IM) injection, followed by intravenous (IV) administration—separated by a 45-day washout period. Pharmaceutical-grade tramadol hydrochloride (Tramal^®^, Cristália, São Carlos, Brazil, 50 mg mL^−1^) was used for intravenous and intramuscular injections. The IM route was administered first in all animals to ensure safer handling and allow the research team to gain experience before performing the more technically demanding IV procedure. To reduce potential bias, all drug administrations were performed by the same experienced veterinarian, and the washout period was deemed sufficient to eliminate any residual drug effects.

### 2.4. Blood Collection

The same time of day was recommended for blood collection during the experiment, starting at 6 a.m.

Blood samples were collected from the paravertebral vein using 3 mL syringes attached to 25 G × 0.7 mm hypodermic needles. Sampling times were as follows: 0 (before tramadol injection), 1 (20 min after injection), 2 (40 min after injection), and 3, 4, 5, 6, 7, 8, and 9 (1-, 2-, 4-, 8-, 12-, 18-, and 26-h post-injection). The total blood collected per animal did not exceed 1% of its live weight. Blood was transferred to tubes containing ethylenediaminetetraacetic acid (EDTA) as an anticoagulant and centrifuged at 3000 rpm for 5 min. Plasma supernatant was stored at −70 °C for further analysis.

### 2.5. Drug Analysis

Tramadol was quantified in *Boa constrictor* plasma using liquid chromatography with fluorescence detection (HPLC-FL) and protein precipitation. The analytical curve preparation followed these steps:

1. Tramadol hydrochloride was dissolved in methanol (10 mL) using ultrasonication to achieve a concentration of 200 µg mL^−1^. This stock solution (500 µL) was diluted with methanol to a final concentration of 10 µg mL^−1^.
2. Ondansetron, used as the internal standard, was prepared at 200 µg mL by dilution with methanol.
3. M1 standard was prepared at 1 mg mL^−1^ stock solution in methanol and then diluted to 5 µg mL^−1^ with methanol.

The mobile phase consisted of a 0.01 M anhydrous disodium phosphate solution with 0.1% triethylamine (*v*/*v*), adjusted to pH 2.9 using phosphoric acid. Calibration standards were prepared by evaporating 30 µL of internal standard in six glass tubes (10 mL each) under a water bath (40 °C) with compressed air. Working standard solutions were then added to achieve tramadol concentrations ranging from 0.1 to 6.0 µg mL^−1^ and M1 concentrations from 0.05 to 2.0 µg mL^−1^. 

Sample Preparation and Chromatographic Conditions: To each tube, 500 µL of blank plasma pool was added. The mixture was vortexed for 1 min, followed by the addition of 2.0 mL of acetonitrile and further vortexing for 1 min. The tubes were then centrifuged at 5000 rpm for 5 min. The resulting supernatant was transferred to clean 10 mL tubes and evaporated to dryness at 40 °C under a stream of compressed air. The dried residue was reconstituted in 500 µL of the mobile phase, vortexed for 1 min, filtered using a syringe filter, transferred to autosampler vials, and subsequently analyzed by high-performance liquid chromatography with fluorescence detection (HPLC-FL). 

Extraction and Mobile Phase Composition: During the protein precipitation step, the final concentration of acetonitrile was 80% (*v*/*v*), resulting from the addition of 2.0 mL of acetonitrile to 500 µL of plasma. The mobile phase used for reconstitution and chromatographic analysis consisted of 75% phosphate buffer (Phase A) and 25% acetonitrile (Phase B). Phase A: 0.01 M anhydrous disodium phosphate aqueous solution containing 0.1% (*v*/*v*) triethylamine, adjusted to pH 2.9 with phosphoric acid.

This mobile phase composition provided an effective elution system for the simultaneous quantification of tramadol, its primary metabolite (M1), and the internal standard (ondansetron) via HPLC-FL.

To simulate the plasma matrix, 500 µL of blank plasma was added to each tube, vortexed for 1 min, followed by the addition of 2.0 mL acetonitrile, and vortexed again. The samples were centrifuged at 5000 rpm for 5 min, and the supernatant was transferred to new tubes and evaporated under airflow at 40 °C. The residue was reconstituted with 500 µL of the mobile phase, filtered through syringe filters, and injected into the HPLC-FL system for analysis. Calibration standards and quality control samples were processed identically.

The tramadol concentration in each sample was determined using the internal standard method, employing peak area ratios and linear regression. HPLC-FL was performed with a mobile phase flow rate of 1 mL min^−1^ and an injection volume of 20 µL.

The development, validation, and quantification of tramadol and its metabolite (O-desmethyltramadol) were conducted at the Quality Control Laboratory of the Pharmacy Faculty, University Federal of Minas Gerais.

### 2.6. Pharmacokinetic Analysis

Pharmacokinetic parameters were calculated using independent equations in Microsoft^®^ Excel 2010. A paired *t*-test was applied to all variables except clearance, which was analyzed using the Wilcoxon test.

Key pharmacokinetic indices included peak concentration (Cmax), time to peak concentration (Tmax), and area under the concentration–time curve (AUC). The elimination half-life (t½) was calculated using the equation t½ = 0.693/λ, where λ is the elimination rate constant. Other indices, such as area under the curve from zero to infinity (AUC_0 → ∞_), mean residence time (MRT_0 → ∞_), volume of distribution (Vd), clearance (CL), and bioavailability (F%), were determined using equations from Shargel & Wu-Pong [9] and Baggot & Giguère [10].

### 2.7. Method Validation Parameters

The suitability of the chromatographic system was evaluated by injecting, in quintuplicate, a methanolic solution containing 10.6 µg/mL of tramadol, 5.0 µg/mL of M1 (O-desmethyltramadol), and 200.0 µg/mL of the internal standard (IS). The average relative peak area of each analyte concentration (expressed as DPR—peak area of each analyte divided by the peak area of the internal standard) was assessed.

Internal standardization was performed by adding a constant, known amount of internal standard to both blank and experimental samples in order to correct for analytical errors related to potential losses of the analytes of interest.

The validation of the bioanalytical method was carried out in accordance with the guidelines outlined in ANVISA Resolution RDC No. 27, dated 17 May 2012 (2012, Brazil), and the European Medicines Agency (EMA) Guideline on Bioanalytical Method Validation (2011, EMA). The parameters evaluated included selectivity, linearity, precision and accuracy, limit of detection (LOD), limit of quantification (LOQ), and short-term stability of the analytes in the biological matrix. The assessment of these parameters ensures the method produces reliable and reproducible results. All data were analyzed using Microsoft Excel^®^ software.

The response for tramadol was considered linear with an R^2^ ≥ 0.99. The method achieved an LOD of 0.03 µg mL^−1^ for M1 and LOQ values of 0.1 µg mL^−1^ for tramadol and 0.05 µg mL^−1^ for M1.

Intra-day accuracy ranged from 97% to 99%, with intra-day precision (RSD%) between 0.5% and 9.1% at concentrations of 0.1 and 5.0 µg mL^−1^. Inter-day accuracy varied between 95% and 98%, with inter-day precision of 8% and 5% for the same concentrations.

The chromatographic conditions used for the HPLC-FL method are listed in Table 1.

### 2.8. Statistical Analysis

A linear mixed-effects model was used to evaluate the effects of administration route and time on plasma concentrations of tramadol and its active metabolite, O-desmethyltramadol (M1). In this model, time, administration route, and their interaction were included as fixed effects, while individual animal was modeled as a random effect to account for intra-animal variability due to repeated measures. Post-hoc comparisons between administration routes (IV vs. IM) were performed separately for tramadol and M1 at each individual time point using Student’s *t*-tests. Likewise, comparisons among different time points within each administration route were also conducted using *t*-tests in order to evaluate temporal variations in plasma concentrations.

Descriptive statistics were expressed as mean ± standard deviation for all variables. Statistical significance was set at *p* < 0.05, and all analyses were performed using R version 4.3.0 (R Core Team, Vienna, Austria, 2023 [11]).

## 3. Results

No adverse effects were observed upon inspection, and the animals’ feeding histories remained normal after the experiment. None of the animals were excluded from the study.

The technique was optimized to provide correlation coefficients (R^2^) of 0.9992 for tramadol and 0.9978 for M1, with detection limits of 0.03 µg mL^−1^ for tramadol and 0.05 µg mL^−1^ for M1, and a limit of quantification of 0.1 µg mL^−1^ for tramadol. Intra-day accuracy (% of nominal concentration) was 97% and 99% for 0.1 and 5 µg mL^−1^, respectively. The intraday precisions (% relative SD) were 9.1% and 0.5%, respectively. Inter-day accuracy (% of nominal concentration) was 95% and 98% for 0.1 and 5 µg mL^−1^, respectively, and inter-day precision was 8% and 5% for 0.1 µg mL^−1^ and 5 µg mL^−1^, respectively.

**Figure 1:**
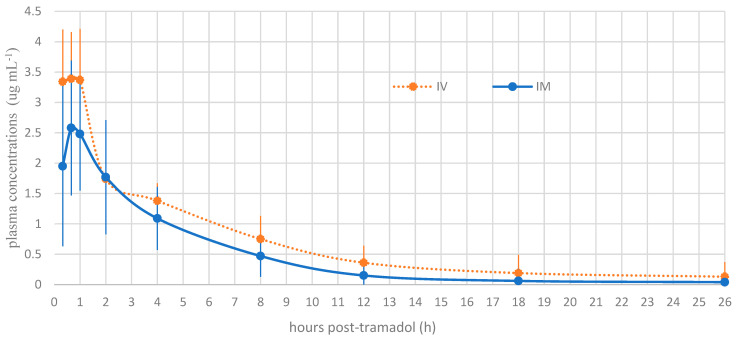
Mean plasma concentration profile of tramadol versus time for intravenous tramadol administration and intramuscular tramadol administration at a dose of 5 mg kg^−1^ in *Boa constrictors* (*n* = 10).

**Figure 2:**
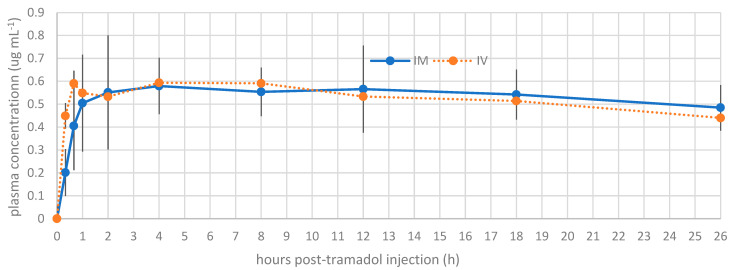
Mean plasma concentration profile of metabolite (M1) versus time for intravenous tramadol administration and intramuscular tramadol administration at a dose of 5 mg kg^−1^ in *Boa constrictors* (*n* = 10).

The plasma concentrations of tramadol and M1 at each time point are presented in Figure 1 and Figure 2, respectively. A statistically significant difference was noted between the intramuscular and intravenous routes at time points 1 (*p* = 0.001) and 2 (*p* = 0.01). Beyond these time points, no significant differences were detected between the routes of administration.

Tramadol and M1 were detected at the first blood collection of the snakes, 20 min after tramadol administration. The different pharmacokinetic parameters of 5 mg kg^−1^ tramadol are detailed in Table 2. The summary of M1 preliminary pharmacokinetic parameters is presented in Table 3. At time T2, corresponding to 40 min after tramadol injection, the observed Cmax values were 3.39 µg/mL and 2.58 µg/mL following IV and IM administration, respectively. For the active metabolite M1, the Cmax was 0.59 µg/mL for IV and 0.58 µg/mL for IM, observed at 40 min (T2) and 4 h post-injection (T5), respectively. The volume of distribution for tramadol was high (>5 L/kg), suggesting extensive tissue distribution. The bioavailability following IM administration was 61%. The clearance (Cl) values were 0.22 ± 0.05 L/kg/h for IV and 0.36 ± 0.07 L/kg/h for IM. Finally, the elimination half-life (t½) of tramadol was prolonged, with mean values of 17.32 ± 7.55 h for IV and 19.96 ± 8.34 h for IM, while for M1, the elimination times were even longer: 35.66 ± 10.85 h for IV and 49.89 ± 10.8 h for IM, suggesting potential accumulation of the active metabolite after a single administration.

## 4. Discussion

This study provides novel and valuable information on the pharmacokinetics of tramadol and its active metabolite, O-desmethyltramadol (M1), following intravenous (IV) and intramuscular (IM) administration in *Boa constrictor*. A key finding is the confirmation that these snakes are indeed capable of producing the M1 metabolite. The reported parameters provide valuable and novel information for future studies on the analgesic efficacy of tramadol and a more rational design of drug prescriptions. The pharmacokinetic parameters of tramadol and M1 in this study differed from those of other reptile species, such as *Chelonoids carbonara* [12] and *Trachemys scripa* [13]. The reasons for these differences are unclear; however, variations among species, individuals, and analytical methods may have affected the determination of plasma concentrations, thereby influencing the pharmacokinetic calculations [12,14].

Based on the Cmax observed, particularly the achievement and sustained maintenance of high plasma concentrations of tramadol and its active metabolite M1, these findings suggest a promising potential for tramadol as an analgesic in snakes when administered intramuscularly. This inference is further supported by the fact that the measured plasma concentrations of tramadol and M1 in *Boa constrictor* significantly exceeded the minimum effective therapeutic concentrations reported in humans [15,16,17,18].

Our preliminary data indicate that tramadol exhibits a relatively long elimination half-life and lower clearance rates in *Boa constrictor.* Furthermore, it was found that the active metabolite M1 has an even more prolonged presence, with an elimination half-life longer than that of tramadol. These findings are particularly relevant given the scarcity of information on analgesics in reptiles [15,16,17,18].

While the general principles of tramadol pharmacokinetics are understood, the specific parameters in *Boa constrictor* show notable differences when compared to other species, underscoring the importance of species-specific studies [6,13,15,19].

The Cmax of tramadol for analgesia in reptiles has not yet been determined. In humans, the reported effective minimum plasma concentrations of tramadol and M1 are approximately 100–500 ng mL^−1^ and 36–84 ng mL^−1^, respectively [15,16,17,18]. In our study, a single dose of 5 mg kg^−1^ of tramadol administered intravenously produced a Cmax of tramadol of 3.39 μg mL^−1^ (3390 ng mL^−1^) and of M1 of 0.59 μg mL^−1^ (590 ng mL^−1^). After intravenous administration of 5 mg kg^−1^ tramadol in *Chelonoids carbonarra*, a Cmax of 0.65 μg mL^−1^ was reported [12]. For intramuscular administration, it was 0.128 μg mL^−1^, with no antinociceptive effect reported for the intravenous administration of 5 mg kg^−1^ or even at a dose of 10 mg kg^−1^ intramuscularly. In our study, tramadol administered intramuscularly resulted in a Cmax of 2.58 μg mL^−1^ (Table 2).

The pharmacokinetics of tramadol in loggerhead sea turtles (*Caretta caretta*) has already been investigated [6]. The researchers administered intramuscular doses of 5 and 10 mg kg^−1^ and reported drug elimination half-lives of 20.35 ± 21 and 22.67 ± 23 h, respectively, while the elimination half-lives of the primary metabolite, O-desmethyltramadol (M1), were approximately 10.23 and 11.26 h. Notably, at both dosing levels, the plasma concentrations of tramadol and M1 remained detectable for several days without eliciting adverse effects. Moreover, plasma concentrations of tramadol and M1 exceeded 100 ng mL for at least 48–72 h following administration of the higher dose. Researchers also analyzed the concentrations of tramadol and its primary metabolite in African penguins (*Spheniscus demersus*) [19]. In this study, 10 mg kg^−1^ of tramadol was administered orally to 15 birds, and blood was collected several times from 0 to 36 h. In this study, plasma concentrations of tramadol surpassed human therapeutic concentrations of 298–590 ng/mL by reaching a mean of 815 ± 151 ng mL^−1^, and these concentrations were maintained for longer than 12 h in more than half of the birds tested [19]. M1 concentrations remained above human therapeutic concentrations of 39.6–84 ng ml^−1^ through at least 36 h in most birds and reached a mean Cmax of 461 ± 91 ng mL^−1^. These findings underscore the necessity of species-specific pharmacokinetic studies, as they highlight interspecies differences in drug metabolism and emphasize the critical importance of tailoring therapeutic protocols to the specific physiological and metabolic characteristics of the species under consideration [19].

The half-life (t½) of tramadol in reptiles is long, as demonstrated in our study. In our investigation, the t½ was 17.32 ± 7.55 h when administered intravenously and 19.96 ± 8.34 h when administered intramuscularly. These values are comparable to those reported in other studies on red-footed tortoises (*Chelonoids carbonara*) that received tramadol intravenously and intramuscularly at doses of 5 mg kg^−1^ (21.22 ± 8.74 and 32.84 ± 6.41, respectively) [12] and in *Trachemys scripa* that received 10 mg kg^−1^ of tramadol intramuscularly (28.7 ± 15.77) [13]. These values support the theory that tramadol is extensively converted into its metabolites in snakes, resulting in a decrease in the plasma concentration of the original compound [12,13].

The volume of distribution (Vd) In this study was high, 5.6 ± 1.69 L kg^−1^ intravenously and 10.58 ± 2.91 intramuscularly, consistent with other studies in various species [6,12,13]. In the pharmacokinetic analysis of tramadol in humans, a high volume of distribution and AUC were observed simultaneously. This suggests that tramadol penetrates extensively into tissues, reflecting a high Vd, and remains in systemic circulation for an extended period, as indicated by the high AUC. This can be attributed to the intrinsic characteristics of tramadol, such as moderate lipophilicity, plasma protein binding, and slow metabolism in reptiles [3]. Clearance values indicate that tramadol elimination was 0.22 (0.15–0.26) kg h L^−1^ intravenously and 0.36 (0.25–0.40) kg h L^−1^ intramuscularly, indicating slow drug elimination. This was expected, given the slower metabolism and lower glomerular filtration rates of these animals [13]. In addition, their urine is not as concentrated as that of mammals because of the inefficiency of water reabsorption mechanisms in the renal tubules [13]. Clearance for M1 was not determined due to insufficient data to accurately model its elimination kinetics. The absence of clearance data for M1 constitutes a recognized limitation of the study, resulting from the need to prioritize animal health and welfare by limiting the total volume of blood collected and, consequently, the pharmacokinetic observation period for the metabolite.

The mean residence time of tramadol was 12.38 ± 8.3 h following intravenous administration and 9.29 ± 5.24 h following intramuscular administration, suggesting that intravenous administration results in prolonged retention of the drug in the body. This extended duration is likely attributable to the reduced metabolic rate observed in these animals [6,13].

The bioavailability after intramuscular administration was 61%. While Giorgi et al. [13] did not determine the bioavailability of tramadol when administering 10 mg kg^−1^ to the hind limb of Trachemy scripta scripta, they obtained higher tramadol and M1 ASC_0 → ∞_ values than in our study. This demonstrates the difference between orders, with Trachemy scripta scripta showing greater tramadol absorption.

The randomization of the administration order was not implemented, as it was determined that the washout period was adequate to eliminate any residual drug effects [13], and the waiting time between administrations was deemed insufficient to introduce bias into the analyses. Administering the drug via the intramuscular route first was a strategic decision to minimize the risk of animal loss during the experiment, given the increased difficulty associated with intravenous administration and the limited sample size. This practical decision also ensured that the serial blood collections conducted during the initial treatment served as training for the administration of the second treatment, reducing the likelihood of missing observations for the intravenous route.

Opioid receptors are widely distributed throughout the bodies of animals, and from an analgesic perspective, tramadol proves beneficial due to its dual peripheral and central actions. It is indicated for nociceptive, inflammatory, and pathological pain, as well as for both chronic and postoperative pain when used in conjunction with nonsteroidal anti-inflammatory drugs [3]. Furthermore, intramuscular tramadol may serve as an adjunctive analgesic [20]. To fully understand the clinical implications of these findings, dynamic studies are necessary to correlate the observed pharmacokinetics with the clinical effects in snakes.

Based on the results presented, substantial formation of the active metabolite M1 was observed in the *Boa constrictor*, with intramuscular administration maintaining comparable concentrations to intravenous administration from T3 through the conclusion of the experiment. This metabolite is primarily responsible for the analgesic effects of tramadol [3,20]. Regarding the comparison of tramadol concentrations between the two administration routes, intramuscular administration offers several advantages over intravenous administration, including ease of application, a reduced risk of hematoma, and fewer potential administration errors. Tramadol appears to be effective when administered intramuscularly in snakes.

While these pharmacokinetic results presented herein for *Boa constrictor* are highly encouraging and align with established therapeutic levels in other species, it is important to emphasize that direct clinical efficacy studies are still needed to confirm these potential benefits in *Boa constrictor*.

## 5. Conclusions

In this study, the pharmacokinetic parameters and absolute bioavailability of tramadol administered intramuscularly in snakes were determined using a noncompartmental approach. Thus, regardless of the route of administration (IV or IM) in snakes, tramadol is rapidly biotransformed into M1, resulting in high concentrations of tramadol over an extended period.

Intramuscular administration of tramadol at a dose of 5 mg kg^−1^ presents itself as a promising option for analgesic management in snakes. This is supported by the rapid biotransformation of tramadol into M1 and the quantification of M1, the main opioid analgesic, at high concentrations in the plasma of the *Boa constrictor*. Furthermore, these high M1 concentrations demonstrated statistical equivalence between the IM and IV routes after 1 h of administration, aligning with concentrations known to be therapeutically relevant in other species. However, it is crucial to note that clinical studies correlating these pharmacokinetic findings with specific snake pharmacodynamics (i.e., direct analgesic efficacy) are necessary to fully validate this potential in a clinical setting.

## Figures and Tables

**Table 1:** The chromatographic conditions of the analytical method HPLC-FL.

Conditions	Description
Analytical column	Phenyl-2 Hypersil (150 × 4.6 mm, 5 µm)
Mobile phase	0.01 M Disodium phosphate dibasic anhydrous:Acetonitrile (75:25 *v*/*v*) 0.1% triethylamine (*v*/*v*) Phosphoric acid (pH 2.9)
Flow rate mobile phase	1 mL min^−1^
Elution mode	Isocrátic
Temperature (°C)	40 °C
Injection volume	20 µL
Detection	202 Excitation wavelength of tramadol 305 Excitation wavelength of ondansetron 296 Emission wavelength of tramadol 365 Emission wavelength of ondansetron 202 Excitation wavelength of M1 296 Emission wavelength of M1
Peaks	M1 3.5 min Tramadol 6.1 min Ondansetron 8.9 min (ID)

**Table 2:** Pharmacokinetic parameters of tramadol, mean ± standard deviation, at a dose of 5 mg kg^−1^ after intravenous and intramuscular administration in *Boa constrictors*.

Parameters	Intravenous	Intramuscular
Cmax (µg mL^−1^)	3.39	2.58
AUC_0 → last_ (µg h mL^−1^)	18.04 ± 4.44	11.95 ± 3.05
t½ (h)	17.32 ± 7.55	19.96 ± 8.34
Vd (L kg^−1^)	5.60 ± 1.69	10.58 ± 2.91
CL (L h kg^−1^)	0.22 ± 0.05	0.36 ± 0.07
MRT_0 → ∞_	12.38 ± 8.30	9.29 ± 5.24
F (%)	-	61

Cmax: maximum concentration; AUC_0 → last_: area under the concentration curve from time zero to last time; t½: elimination half-life; Vd: volume of distribution; CL: clearance; MRT_0 → ∞_: mean residence time from zero to infinity; F %: bioavailability.

**Table 3:** Pharmacokinetic parameters of O-desmethyltramadol (M1), mean ± standard deviation, at a dose of 5 mg kg^−1^ after intravenous and intramuscular administration in *Boa constrictors*.

Parameters	Intravenous	Intramuscular
Cmax (µg mL^−1^)	0.59	0.58
AUC_0→last_ (µg h mL^−1^)	13.68 ± 5.04	13.60 ± 2.76
t½ (h)	35.66 ± 10.85	49.89 ± 10.8
MRT_0 → ∞_ (h)	77.46 ± 0.74	97.99 ± 3.84

Cmax: Maximum concentration; AUC_0 → last_: area under the concentration curve from time zero to last time; t½: elimination half-life; MRT_0 → ∞_: mean residence time from zero to infinity.

## Data Availability

The data presented in this study are openly available in [Repositório Institucional da UFMG] at [http://hdl.handle.net/1843/60844, accessed on 5 August 2025].
